# Identifying novel β-lactamase substrate activity through *in silico* prediction of antimicrobial resistance

**DOI:** 10.1099/mgen.0.000500

**Published:** 2021-01-08

**Authors:** Kara K. Tsang, Finlay Maguire, Haley L. Zubyk, Sommer Chou, Arman Edalatmand, Gerard D. Wright, Robert G. Beiko, Andrew G. McArthur

**Affiliations:** ^1^​ David Braley Centre for Antibiotic Discovery, McMaster University, Hamilton, Ontario, Canada; ^2^​ M.G. DeGroote Institute for Infectious Disease Research, McMaster University, Hamilton, Ontario, Canada; ^3^​ Department of Biochemistry and Biomedical Sciences, McMaster University, Hamilton, Ontario, Canada; ^4^​ Faculty of Computer Science, Dalhousie University, Halifax, Nova Scotia, Canada

**Keywords:** antimicrobial resistance, bioinformatics, genotype–phenotype, prediction

## Abstract

Diagnosing antimicrobial resistance (AMR) in the clinic is based on empirical evidence and current gold standard laboratory phenotypic methods. Genotypic methods have the potential advantages of being faster and cheaper, and having improved mechanistic resolution over phenotypic methods. We generated and applied rule-based and logistic regression models to predict the AMR phenotype from *Escherichia coli* and *Pseudomonas aeruginosa* multidrug-resistant clinical isolate genomes. By inspecting and evaluating these models, we identified previously unknown β-lactamase substrate activities. In total, 22 unknown β-lactamase substrate activities were experimentally validated using targeted gene expression studies. Our results demonstrate that generating and analysing predictive models can help guide researchers to the mechanisms driving resistance and improve annotation of AMR genes and phenotypic prediction, and suggest that we cannot solely rely on curated knowledge to predict resistance phenotypes.

## Data Summary

All genomic data analysed in this work are available through National Center for Biotechnology Information (NCBI) BioProject PRJNA532924. All conda environments, code and intermediate data files required to generate this analysis are available at: https://github.com/karatsang/rulesbased_logisticregression, https://doi.org/10.5281/zenodo.3988480.

**Impact Statement**

Antimicrobial resistance (AMR) is an increasingly global crisis and there is need for technologies that can diagnose and surveil it. We compare statistical modelling and rules-based approaches to predict AMR phenotypes for *Escherichia coli* and *Pseudomonas aeruginosa* clinical isolates based on genome sequence. With an emphasis on the substrate activities of clinically important β-lactamases, our algorithms predict previously unknown β-lactamase substrate activities. We validate these novel substrate activities using a robust experimental target gene expression system. Our work illustrates that known clinical AMR gene threats have a broader range of antibacterial activity than previously thought, with important implications for antibiotic stewardship.

## Introduction

Antimicrobial resistance (AMR) is a global health crisis accelerated by overuse and misuse of antimicrobials. Amongst Gram-negative pathogens, AMR *Escherichia coli* and *Pseudomonas aeruginosa* are of urgent and critical concern. The World Health Organization has reported high resistance to fluoroquinolones and third-generation cephalosporins when treating urinary tract *E. coli* infections, leading to reliance on carbapenems as a last-resort treatment option [1], while the US Centers for Disease Control and Prevention estimates nearly 32 600 antibiotic-resistant *P. aeruginosa* infection-related hospitalizations in the USA alone in 2017, to which 2700 deaths were attributed [2].

Currently, the gold standards for diagnosing antibiotic resistance are culture-based phenotypic methods. However, the turnaround time for antibiotic susceptibility tests often surpasses the optimal time for life-threatening infection treatment [3, 4]. Furthermore, phenotypic tests do not reveal the genetic underpinnings of resistance. As such, genotypic methods that exploit high-throughput DNA sequencing technology combined with bioinformatics resources have the potential to be faster and more accurate and informative than the current phenotypic paradigm [5]. There is growing momentum toward whole-genome sequencing of clinical infections, but there is a lag in the development of bioinformatic platforms that can accurately predict phenotypes such as virulence and AMR, which is essential for the full application of rapid pathogen sequencing as a robust diagnostic tool. Most sequencing pipelines rely on an AMR sequence database to predict functional AMR genes from DNA sequences [6], of which there are many. For example, the Comprehensive Antibiotic Resistance Database (CARD) is an ontology-driven genomics database used by the Resistance Gene Identifier (RGI) software to predict intrinsic and acquired resistance determinants in genome sequences [7]. The Antibiotic Resistance Gene-ANNOTation database [8] and Pathosystems Resource Integration Center [9] store a similar breadth of resistance determinants to CARD and also use blast-based tools for resistome annotations. Antibiotic Resistance Genes Online [8] only catalogues β-lactam and vancomycin resistance determinants, in comparison to ResFinder [10], which primarily annotates acquired resistance genes using blastn, while ResFams [11] is a database of protein domain hidden Markov models associated with AMR function.

Despite our dependence upon curated AMR databases for genotype analysis and prediction of phenotype, maintaining and developing AMR databases and tools are challenging due to the ever evolving AMR genetic landscape, inconsistencies in AMR gene nomenclature, sparsity of phenotypic data and lack of funding for biocuration [12, 13]. Without comprehensiveness in phenotypic testing, such as antibiotic susceptibility testing using a broad panel of antibiotics, all of these databases will inherently be missing the full range of a resistance determinant’s substrate specificity. Yet, as β-lactams are the most commonly used antibiotic [14], there is strong motivation in the AMR field to identify the substrate specificity of clinically prevalent β-lactamases [14–20], particularly with regard to β-lactams new to the marketplace. Despite the development of gene-based antibiotic susceptibility testing tools such as the Antibiotic Resistance Platform [21], when novel β-lactamases emerge in clinical settings they are often only characterized using a limited selection of β-lactams, or are assumed to have similar substrate activity to a related β-lactamase. This leads to knowledge gaps in AMR databases for β-lactamase substrate specificity. In the face of missing experimental data, the prediction of novel substrate specificities for known β-lactamases can be performed using statistical modelling and machine learning methods [22–24]. While these statistical models can be used to discover novel genotype–phenotype relationships, they often require large and diverse datasets to be effective. Previous studies have used rule-based and statistical models to predict antibiotic resistance phenotypes from genotypes, but only a few studies provide genotype–phenotype associations [22, 24].

Here we report the *in silico* prediction of genotype–phenotype associations and substrate specificities for AMR determinants from multidrug-resistant *E. coli* and *P. aeruginosa* clinical isolates using two computational approaches (rules-based and logistic regression) based upon CARD’s RGI [7]. The rules-based method uses new software (the Efflux Pump Identifier) to account for overexpressed multi-component efflux pumps as well as hand-curated knowledge encoded by CARD’s Antibiotic Resistance Ontology (ARO). This method helped identify that gaps in CARD’s curated knowledge of β-lactam substrate activity contributed to poor β-lactam resistance phenotype prediction. We then performed logistic regression on the same data, observing higher prediction accuracy across most antibiotic resistance phenotypes. We were then able to experimentally validate the predicted genotype–phenotype relationships (i.e. learned weights) used by logistic regression to identify previously unknown β-lactamase substrate activities.

## Results

### Bacterial isolates, antibiotic susceptibility testing (AST), and whole-genome sequencing

In total, 115 *E. coli* and 102 *P *. *aeruginosa* putative multidrug-resistant clinical isolates were obtained from Hamilton Health Sciences hospitals (Hamilton, Ontario, Canada) and submitted for both genome sequencing and AST, i.e. categorized as ‘resistant’ or ‘susceptible’ for 18 antibiotics under Clinical and Laboratory Standards Institute (CLSI) guidelines. Among the isolates, 20 *E. coli* had no resistance to any of the tested antibiotics and all of the *P. aeruginosa* strains were resistant to at least 1 drug. Seventy-four *E. coli* and 101 *P *. *aeruginosa* isolates were resistant to 3 or more antibiotics. The antibiotics tested and the full AST results are summarized in https://github.com/karatsang/rulesbased_logisticregression/tree/v1.0.0/AST. In the *E. coli* dataset there were 30 unique multilocus sequence types (MLSTs) and 5 isolates with unresolved MLST allele(s). The 2 most prevalent *E. coli* MLSTs in the dataset were ST131 and ST1193, which 39 and 10 clinical isolates belonged to, respectively. Notably, ST131 is known to be a major cause of multidrug-resistant *E. coli* infections in the USA [25] and a globally dominant clone [26] associated with CTX-M β-lactamases, while ST1193 is a newer multidrug-resistant *E. coli* clonal group (2017–2019) associated with both CTX-M β-lactamases, plasmid-borne TEM-1 and aminoglycoside acetyltransferases (AACs) [27–29]. In the *P. aeruginosa* dataset there were 59 unique MLSTs (43 known and 16 novel MLSTs) and 3 isolates with unresolved MLST allele(s). The three most prevalent MLSTs, ST244, ST235 and ST253, were identified in five *P *. *aeruginosa* isolates each. *P. aeruginosa* ST244 is an international clone, many isolates of which are multidrug-resistant [30, 31], ST235 is amongst the most prevalent of international clones originating from Europe, with regional acquisition of AMR genes [32], and ST253 a less common clone associated with multidrug resistance in Spain and Greece [33]. The full MLST results are summarized in https://github.com/karatsang/rulesbased_logisticregression/tree/v1.0.0/MLST. Raw Illumina DNA sequencing reads for each isolate are available through National Center for Biotechnology Information (NCBI) BioProject PRJNA532924.

### Rules-based interpretation leads to poor β-lactam phenotype prediction

Our rules-based algorithm relies on the resistome predictions of CARD’s RGI and the genotype–phenotype relationships curated in CARD’s ARO. RGI uses four bioinformatics models to predict the resistome of a clinical isolate, which are the protein homology, protein variant, rRNA variant and protein overexpression models (detailed at https://github.com/arpcard/rgi). The protein homology model detects a protein sequence based on its similarity to a curated reference sequence in CARD. The protein variant model builds on the protein homologue model to identify curated mutations that are shown to confer resistance in antibiotic targets, while the rRNA variant model performs the same function for mutations conferring resistance to antibiotics targeting ribosomal RNAs. The protein overexpression model identifies proteins with or without mutations which reflects regulatory proteins that are functional without a mutation, but confer overexpression of their targets with a mutation. As CARD’s RGI software is unable to predict multi-component efflux pump systems important for AMR, we developed the Efflux Pump Identifier (EPI) software to interpret RGI results for the prediction of overexpressed efflux pump systems, classifying them into three categories: Perfect, Partial and Putative. The Perfect category identifies sequence matches to CARD for all components of a predicted efflux multi-component system. The Partial category identifies all components of an efflux multi-component system, but at least one component is a sequence variant of CARD’s reference sequence. The Putative category predicts potential efflux multi-component systems with missing components or otherwise entirely composed of previously uncharacterized sequence variants.

For our analyses we used the above models and RGI’s Perfect and Strict criteria, supplemented with the EPI’s interpretation of efflux complexes, to predict resistomes from isolate genome sequences. RGI’s Perfect criterion requires that a query protein sequence be identical to a curated reference sequence in CARD, while Strict detects variants of known resistance determinants that pass a curated bit-score cut-off (protein homologue model) or a known AMR-conferring mutation (protein variant model) that can be found curated within CARD (card.mcmaster.ca). The predicted resistomes of the individual *P. aeruginosa* and *E. coli* isolates were generally unique and contained a large diversity of resistance determinants (Table 1, also see https://git.io/JJFh3), with the exceptions being two groups of three *P. aeruginosa* isolates and five *E. coli* isolates that had the same predicted resistome, respectively.

**Table 1.** The prevalence of Perfect and Strict resistance determinants detected by the Resistance Gene Identifier, organized by the Antibiotic Resistance Ontology (ARO) drug class designations. Columns show the number and percentage of sampled isolates with at least one AMR determinant associated with resistance to each drug class, broken down as harbouring efflux or non-efflux determinants, or both. For example, 98 % of all *P. aeruginosa* isolates had a least one resistance gene for rifamycin resistance, with 99 isolates predicted to have only efflux gene(s) conferring resistance to rifamycin and a single isolate predicted to have only a non-efflux determinant of rifamycin resistance. The total number of *E. coli* and *P. aeruginosa* isolates is 115 and 102, respectively.

ARO drug class	No. of *E. coli* isolates (non-efflux+efflux+both)	% of *E. coli* isolates	No. of *P. aeruginosa* isolates (non-efflux+efflux+both)	% of *P. aeruginos *a isolates
Acridine dye	0+115+0	100.0 %	0+102+0	100.0 %
Aminocoumarin antibiotic	0+114+1	100.0 %	0+101+1	100.0 %
Aminoglycoside antibiotic	0+44+71	100.0 %	0+0+102	100.0 %
Benzalkonium chloride	0+115+0	100.0 %	0+1+0	1.0 %
Bicyclomycin	0+1+0	0.9 %	0+102+0	100.0 %
Carbapenem	0+0+115	100.0 %	0+0+102	100.0 %
Cephalosporin	0+0+115	100.0 %	0+0+102	100.0 %
Cephamycin	0+0+115	100.0 %	0+101+1	100.0 %
iaminopyrimidine antibiotic	50+1+3	47.0 %	0+101+1	100.0 %
Elfamycin antibiotic	115+0+0	100.0 %	2+0+0	2.0 %
Fluoroquinolone antibiotic	0+42+73	100.0 %	0+67+35	100.0 %
Fosfomycin	0+111+4	100.0 %	102+0+0	100.0 %
Fusidic acid	0+1+0	0.9 %	0+0+0	0.0 %
Glycopeptide antibiotic	0+111+4	3.5 %	2+0+0	2.0 %
Glycylcycline	0+115+0	100.0 %	0+100+0	98.0 %
Lincosamide antibiotic	4+68+3	65.2 %	3+1+0	3.9 %
Macrolide antibiotic	0+60+55	100.0 %	0+0+102	100.0 %
Monobactam	0+0+115	100.0 %	0+0+102	100.0 %
Mupirocin	0+0+0	0.0 %	1+0+0	1.0 %
Nitrofuran antibiotic	115+0+0	100.0 %	0+2+0	2.0 %
Nitroimidazole antibiotic	0+115+0	100.0 %	0+0+0	0.0 %
Nucleoside antibiotic	0+112+3	100.0 %	0+1+0	1.0 %
Nybomycin	72+0+0	62.6 %	21+0+0	20.6 %
Oxazolidinone antibiotic	0+0+0	0.0 %	1+0+0	1.0 %
Penam	0+0+115	100.0 %	0+0+102	100.0 %
Penem	0+65+50	100.0 %	0+99+3	100.0 %
Peptide antibiotic	0+0+115	100.0 %	0+0+0	100.0 %
Phenicol antibiotic	0+91+24	100.0 %	0+1+101	100.0 %
Pleuromutilin antibiotic	39+0+0	33.9 %	1+0+0	1.0 %
Rhodamine	0+115+0	100.0 %	0+1+1	1.0 %
Rifamycin antibiotic	0+115+0	100.0 %	0+99+1	98.0 %
Streptogramin antibiotic	42+0+0	36.5 %	3+0+0	2.9 %
Sulfonamide antibiotic	67+0+0	58.3 %	0+94+8	100.0 %
Sulfone antibiotic	67+0+0	58.3 %	8+0+0	7.8 %
Tetracycline antibiotic	0+112+3	100.0 %	0+99+3	100.0 %
Triclosan	0+114+1	100.0 %	0+102+0	100.0 %

In the *P. aeruginosa* clinical isolate dataset, RGI detected 4 Perfect and 38 Strict, non-efflux, unique resistance genes (protein homologue models) across 34 of CARD’s drug classes, plus 4 unique, non-efflux mutations (protein variant models) known to confer resistance to particular antibiotics (ParE A473V, GyrA T83I, BasR L71R and EF-Tu R234F). In the *E. coli* dataset, RGI detected 31 Perfect and 59 Strict non-efflux, unique resistance genes (protein homologue models), plus 15 unique, non-efflux mutations or combinations of mutations (protein variant models) known to confer resistance to particular antibiotics (UhpT E350Q; ParC S80I, E84G; EF-Tu R234F; PBP3 D350N, S357N; GlpT E448K; GyrB S464Y; GyrA D87Y, D87G, D87N, S83L; CyaA S352T; PtsI V25I; NfsA Y45C). For efflux, in *P. aeruginosa* there were 2 unique Perfect and 14 Strict and in *E. coli* there were 11 unique Perfect and 34 Strict protein homologue models representing single-component efflux resistance genes. EPI additionally detected one Perfect or Partial efflux complex with an overexpression mutation (* E. coli* AcrAB-TolC with MarR mutation Y137H conferring resistance to ciprofloxacin and tetracycline) in two different *E. coli* isolates; otherwise, EPI identified six unique Partial efflux pump complexes without an overexpression mutation among the *E. coli* isolates. In contrast, EPI did not identify any Perfect efflux pump complexes among *P. aeruginosa* isolates; however, three unique Partial efflux pump complexes with an overexpression mutation were identified in three different clinical isolates (MexEF-OprN with MexS F253L,V73A; MexAB-OprM with MexR R91C; MexAB-OprM with NalC S209R, G71E, A186T). Supplementary information and citations for all variants predicted by RGI/EPI can be found at CARD.

**Fig. 1.**
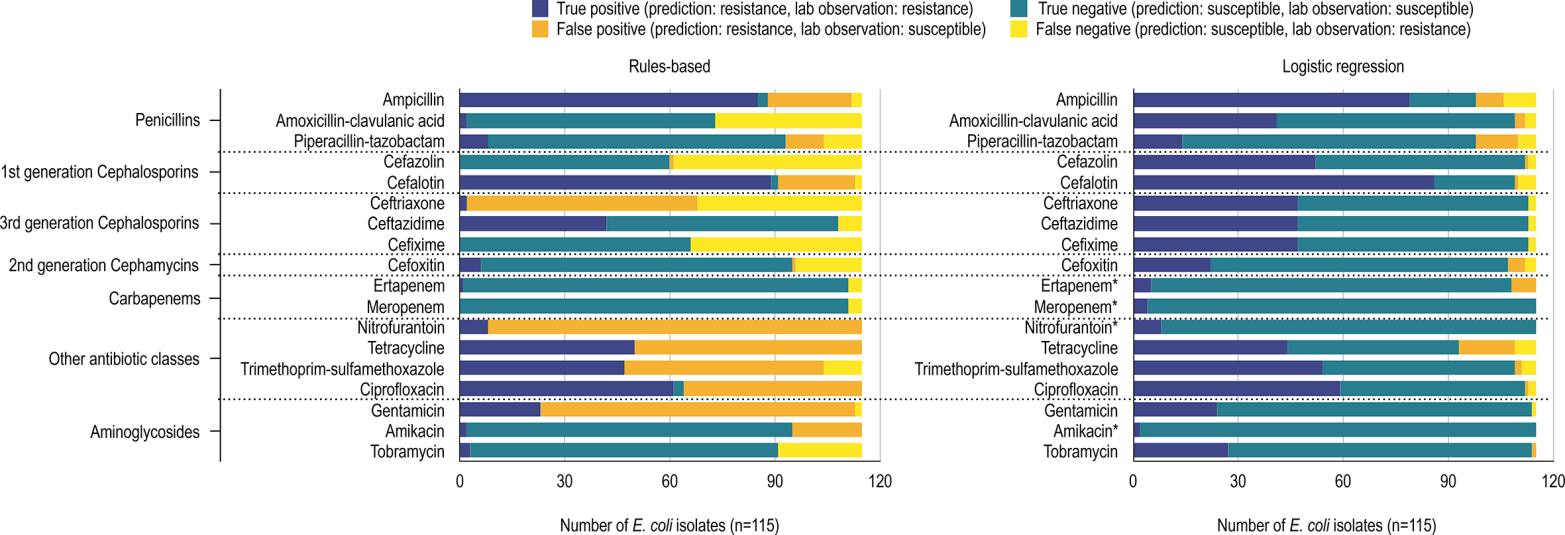
True-positive, true-negative, false-positive and false-negative predictions of *E. coli* resistance phenotype using a rules-based (left) and logistic regression (right) method. Antibiotic susceptibility tests used 18 antibiotics organized into their respective drug classes. True positives (dark blue) and true negatives (teal) indicate that the classifier predicted resistance and susceptibility correctly. False positives (orange) indicates classifier prediction of resistant but an AST of susceptible. Similarly, false negatives (yellow) indicates classifier prediction of susceptible but an AST of resistant. The rules-based method uses RGI, EPI and the Antibiotic Resistance Ontology to predict resistance phenotypes. Logistic regression classifiers use RGI-detected AMR determinants to predict resistance phenotypes. Logistic regression models for antibiotics for which <10 % of a species’ isolates displayed susceptible or resistant phenotypes could not be properly validated and tested and as such were trained using all the data (indicated by an asterisk).

**Fig. 2.**
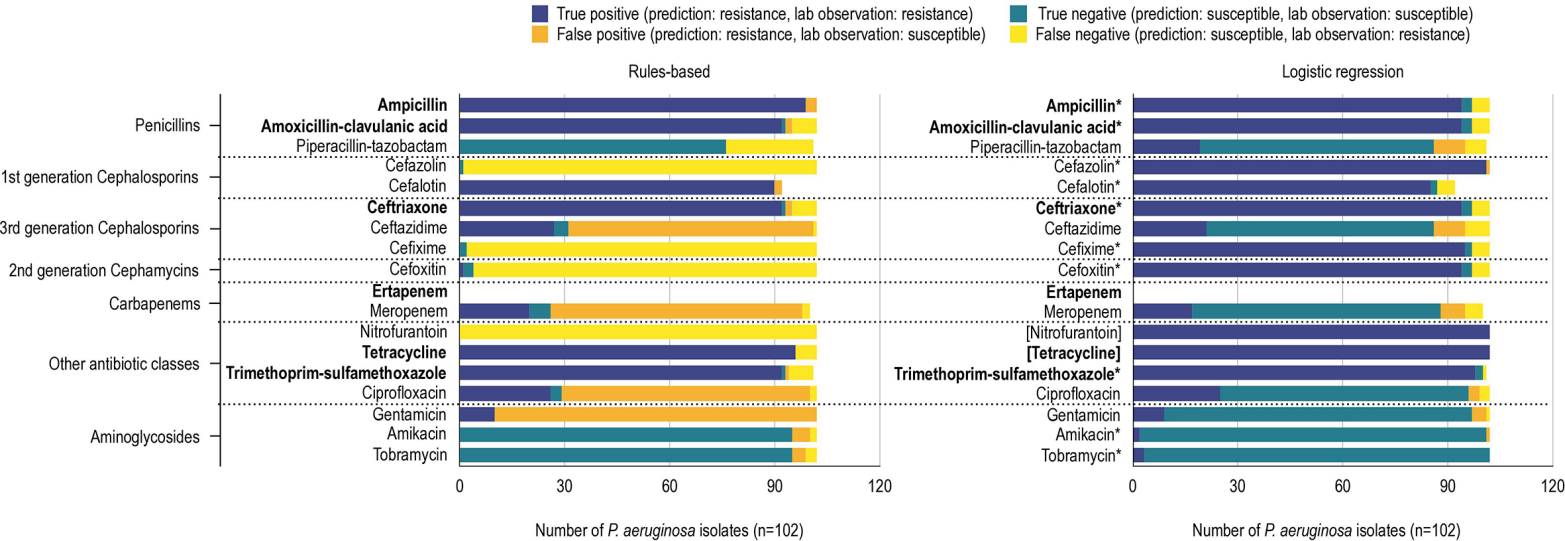
True-positive, true-negative, false-positive and false-negative predictions of *P. aeruginosa* resistance phenotype using a rules-based (left) and logistic regression (right) method. Antibiotic susceptibility tests used 17 antibiotics (ertapenem was not tested in *P. aeruginosa *) organized into their respective drug classes. Prediction performances for antibiotic logistic regression classifiers using RGI detected AMR determinants to predict resistance phenotypes for *E. coli* and *P. aeruginosa *. True positives (dark blue) and true negatives (teal) indicate that the classifier predicted resistance and susceptibility correctly. False positives (orange) indicates classifier prediction of resistant but an AST of susceptible. Similarly, false negatives (yellow) indicates classifier prediction of susceptible but an AST of resistant. The rules-based method uses RGI, EPI and the Antibiotic Resistance Ontology to predict resistance phenotypes. Logistic regression classifiers use RGI-detected AMR determinants to predict resistance phenotypes. Logistic regression models for antibiotics for which <10 % of a species’ isolates displayed susceptible or resistant phenotypes could not be properly validated and tested and as such were trained using all the data (indicated by an asterisk). Similarly, when all isolates were resistant or susceptible a ‘dummy’ model was used, which always returns the relevant label (placed in square brackets). The bolded antibiotics represent antibiotics that *P. aeruginosa* confer intrinsic resistance towards, according to the Clinical and Laboratory Standards Institute (CLSI). The total of *P. aeruginosa* phenotype predictions does not always equal the total number of isolates (*n*=102) because not all isolates were tested against every antibiotic.

Comparing the above RGI and EPI resistome predictions, phenotypically classified by CARD’s ARO, to the laboratory ASTs, we observed instances of true-positive, true-negative, false-positive and false-negative predictions of AMR phenotype for both *E. coli* and *P. aeruginosa* (Figs 1 and 2).

No antibiotic resistance phenotypes were predicted with 100 % accuracy (defined as the percentage of correctly classified phenotypes). Most of the penicillin and cephalosporin (amoxicillin/clavulanic acid, piperacillin/tazobactam, cefazolin, ceftriaxone, ceftazidime, cefixime and meropenem) resistance phenotype predictions resulted in false negatives for both *E. coli* and *P. aeruginosa* (i.e. we failed to predict the observed resistance based on genome sequence). In particular, the prediction of both cefazolin and cefixime resistance phenotypes was less than 2 % accurate in the *P. aeruginosa* dataset and less than 57 % accurate in the *E. coli* dataset. In addition, for *E. coli* the rules-based algorithm failed to predict any of the observed cefazolin and cefixime resistance based on genome sequence (i.e. not a single true-positive result was obtained).

### Logistic regression improves AMR phenotype prediction accuracy

A limitation of the rules-based method is that it only uses known and curated information to predict resistance and is thus inherently blind to any unknown AMR genotype–phenotype relationships. To overcome this limitation, we used logistic regression (LR) to independently identify patterns between RGI-predicted AMR determinants and observed AMR phenotypes. For the *E. coli* dataset (*n*=115) it was possible to train LR classification models, optimized via cross-validation, and test them on a set of withheld isolates for 14 out of 18 antibiotics (Fig. 1). Due to the relative imbalance of resistant versus susceptible isolates for amikacin, ertapenem, meropenem and nitrofurantoin, models trained for these antibiotics required the use of all isolates, preventing the evaluation of model generalizability on a held-out test set. In the *P. aeruginosa* dataset, piperacillin/tazobactam, ceftazidime, meropenem, ciprofloxacin and gentamicin resistance prediction models were trained and tested on separate isolates, while nitrofurantoin and tetracycline required use of ‘dummy’ models (i.e. all isolates were intrinsically resistant) and the remainder of the AMR prediction models were trained on all isolates due to unbalanced sampling of resistant and susceptible isolates (Fig. 2).

We evaluated model performance using test set average precision (i.e. trapezoidal area under the precision–recall curve) and a model was categorized as very precise if the test set average precision was ≥0.85, relative to previous studies. Generally, our models were very precise with our *E. coli* data, with a test set average precision of ≥0.85 for all antibiotics except amoxicillin/clavulanic acid (0.811), piperacillin/tazobactam (0.435) and cefoxitin (0.385). In contrast, the *P. aeruginosa* dataset was particularly problematic for LR, with the majority of resistance phenotypes being either ubiquitous (tetracycline and nitrofurantoin) or the less-frequent phenotype representing fewer than 10 % of isolates (10/17 antibiotics; ertapenem was not evaluated for these isolates) (Fig. 2). Only five antibiotics had properly fitted and evaluated models for *P. aeruginosa *: ceftazidime, ciprofloxacin, gentamicin, meropenem and piperacillin/tazobactam. These models had either moderate (ciprofloxacin:~0.650), poor (ceftazidime, piperacillin/tazobactam: 0.512, 0.403), or extremely poor (meropenem: 0.227, gentamicin C: 0.196) test set average precision.

Overall, using LR reduced problems of false-positive and false-negative prediction of AMR phenotypes (Figs 1 and 2). For *P. aeruginosa* cefazolin and cefixime resistance phenotypes, where the rules-based approach had very few accurate predictions, LR was able to improve accuracy by 92 and 98 %, respectively. Similarly, the rules-based method could not predict any true-positive *E. coli* cefazolin and cefixime resistance phenotypes, whereas LR improved accuracy by 45 and 41 %, respectively. In both *P. aeruginosa* and *E. coli* datasets, LR reduced the number of false positives in most tested antibiotic resistance phenotypes compared to the rules-based method. Even in the antibiotic resistance phenotypes where the number of false positives increased, prediction accuracy still improved, e.g. *P. aeruginosa* piperacillin/tazobactam resistance and *E. coli* tobramycin resistance (Figs 1 and 2).

### LR models predict novel β-lactamase activity

**Fig. 3.**
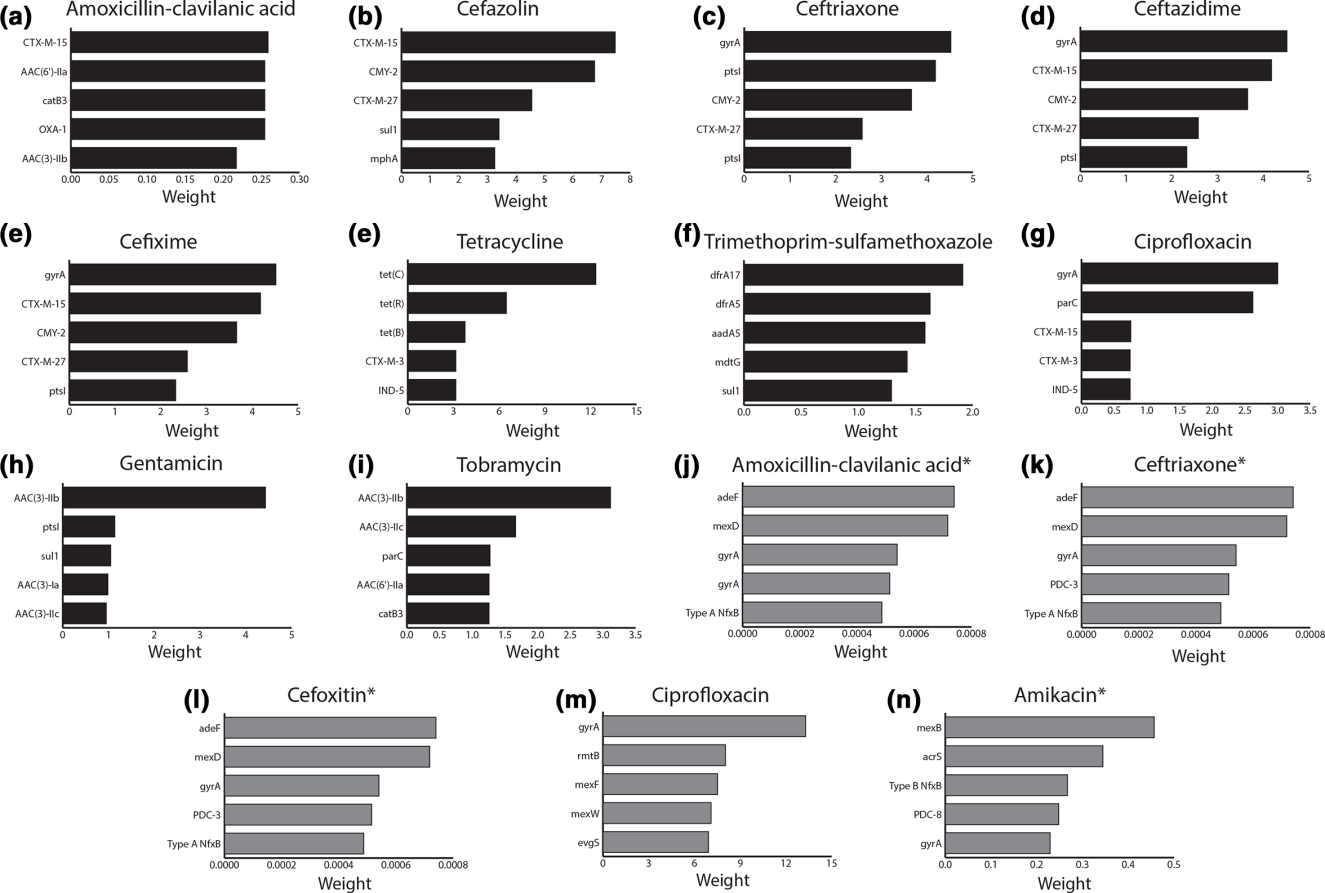
Logistic regression and RGI identify resistance determinants for predicting *E. coli* and *P. aeruginosa* resistance phenotypes that are supported by the literature. The *x*-axes indicate assigned logistic regression weights for individual AMR phenotype predictions, while the *y*-axes list the top five weighted AMR determinants. Black and grey bars represent *E. coli* and *P. aeruginosa* resistance phenotypes, respectively. An asterisk indicates that <10 % of a species’ isolates displayed a susceptible or resistant phenotype to amikacin and therefore could not be properly validated and tested, so were trained using all of the data. Models identifying resistance determinants inconsistent with the literature are shown in Figs S4 and S5.

For every antibiotic resistance phenotype, LR assigns every resistance determinant a weight to estimate its relative contribution to overall resistance. We investigated the five most highly weighted predictors for each antibiotic and pathogen to examine the predicted AMR genotype–phenotype relationships. LR weights that confirmed a known relationship (i.e. supported by the published literature and already curated in CARD) for *E. coli* included *CTX-M-15* for ceftazidime resistance, *tet(C*) for tetracycline resistance, *aac (3)-IIb* for gentamicin and tobramycin resistance, *dfrA17* for trimethoprim/sulfamethoxazole resistance, and *gyrA* for ciprofloxacin resistance (Fig. 3a–j) and for *P. aeruginosa* included *mexD* for amoxicillin/clavulanic acid, ceftriaxone, and cefoxitin resistance, *gyrA* for ciprofloxacin resistance, and *mexB* for amikacin resistance (Fig. 3k–o).

**Fig. 4.**
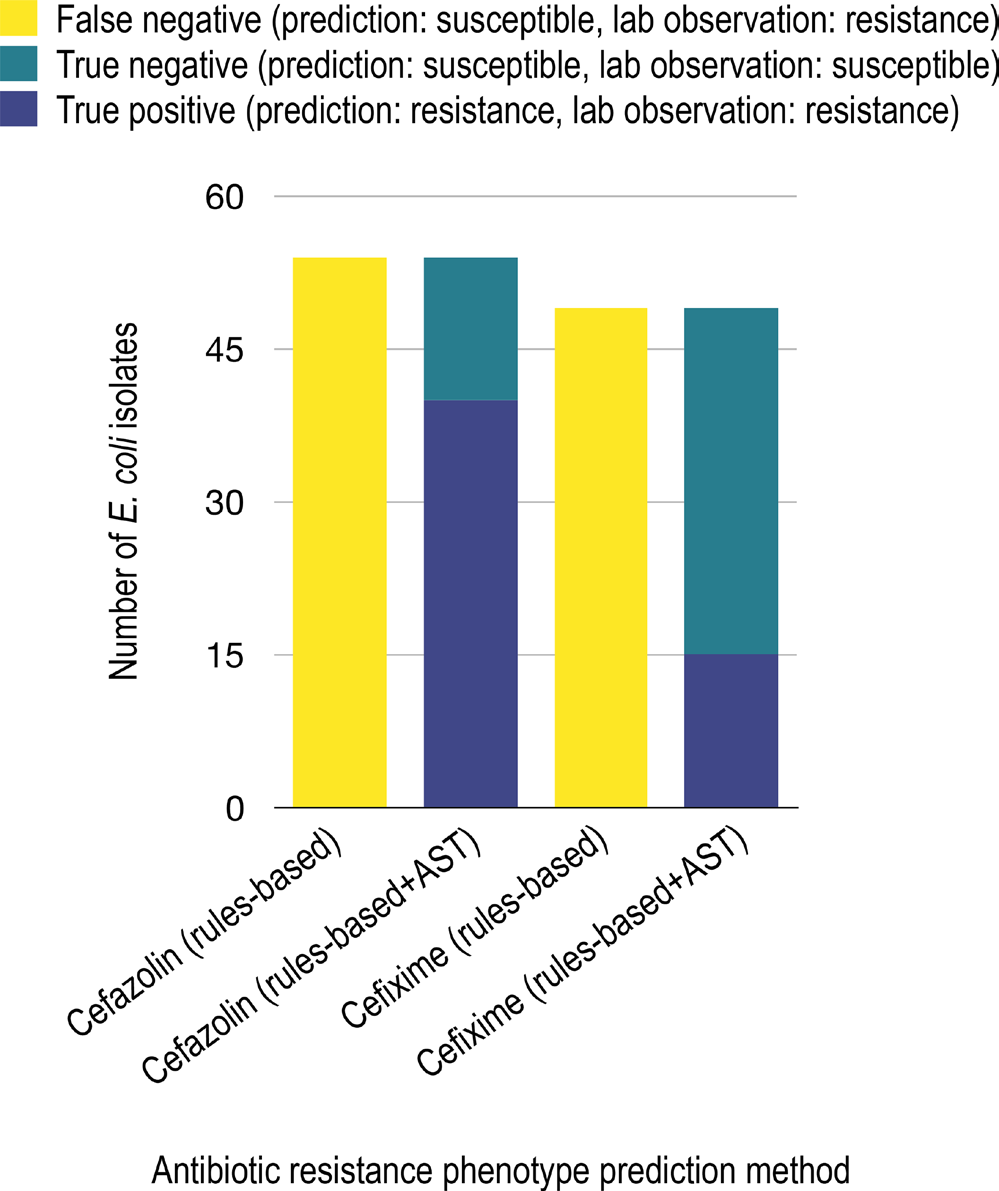
Improvement of *E. coli* cefazolin and cefixime resistance prediction using rules-based algorithm and substrate activity knowledge gained from antibiotic susceptibility testing (AST). Through antibiotic susceptibility testing, we observed CTX-M-3, CTX-M-27 and CMY-2 conferring clinically relevant resistance to cefazolin and cefixime. Curating this knowledge into CARD would improve cefazolin and cefixime true positive resistance prediction in *E. coli* by 74.1 and 30.6 %, respectively.

A number of the most highly weighted predictors suggested a previously undocumented substrate specificity for a known β-lactamase, most notably *CMY-2* conferring resistance to amoxicillin/clavulanic acid and cefazolin, along with *CTX-M-15* conferring resistance to cefixime. To independently test these highly weighted associations, we tested the substrate activity of 11 resistance genes predicted in either the *E. coli* isolates (*aac(6′)-Ib-cr*, *CMY-2*, *CTX-M-15*, *CTX-M-3*, *CTX-M-27*, *OXA-1*, *OXA-50*, *TEM-1* and *TEM-30*) or *P. aeruginosa* isolates (*PDC-3* and *PDC-5*) using the Antibiotic Resistance Platform (ARP) [21], concluding clinical resistance based on a ≥2-fold elevation in minimum inhibitory concentration (MIC) compared to control that also passed the CLSI Resistant MIC breakpoint value. In total, 22 previously unknown activities between 7 AMR genes and an antibiotic were experimentally validated as clinically relevant in at least 1 pathogen using the ARP and CLSI breakpoints (Table 2). These included new knowledge for resistance to ampicillin (*CMY-2*, *CTX-M-3*, *CTX-M-27, OXA-1* and *TEM-30*), amoxicillin/clavulanic acid (*CMY-2*, *CTX-M-3*, *OXA-1* and *TEM-1*), cefazolin (*CMY-2*, *CTX-M-3*, *CTX-M-15*, *CTX-M-27* and *TEM-1*), cefixime (*CMY-2* and *CTX-M-3*), ceftazidime (*CMY-2*, *CTX-M-3* and *CTX-M-27*), ertapenem (*CTX-M-27*) and ceftriaxone (*CMY-2* and *CTX-M-3*). However, none of the tested resistance genes explained the observed resistance to meropenem and an additional four genes only confirmed previous knowledge: AAC(6′)-Ib-cr conferring resistance to tobramycin [34], TEM-1 conferring resistance to ampicillin [35], TEM-30 conferring resistance to amoxicillin/clavulanic acid [36] and CTX-M-15 conferring resistance to ceftriaxone (Table S1, available in the online version of this article) [37]. ASTs also invalidated some predictions, e.g. *CTX-M-15* conferring clinically relevant resistance towards cefixime and ceftazidime. Notably, while *OXA-50* is reported to elevate the MIC towards ampicillin and cefotaxime when cloned into a multicopy plasmid and expressed in *P. aeruginosa,* like others [38], we did not observe any appreciable elevation in MIC compared to control in *E. coli* (data not shown). Overall, LR combined with AST validation provided a wealth of new knowledge on antibiotic specificities for β-lactamases appearing in clinical isolates. Interestingly, incorporation of these results into the rules-based algorithm improved resistance prediction in *E. coli* for cefazolin (75 % improvement in true-positive results) and cefixime (31 % improvement in true-positive results) (Fig. 4) plus in *P. aeruginosa* for cefixime (34 % improvement in true-positive results) and cefoxitin (35 % improvement in true-positive results) (Fig. S6), illustrating the sensitivity of rules-based methods to available knowledge. Yet, even with this new knowledge, the rules-based algorithm was still outperformed by the LR approach.

**Table 2.** Antibiotic susceptibility testing (AST) of known resistance genes predicted to have previously undescribed activity. As per the Antibiotic Resistance Platform, AMR genes were cloned into the pGDP plasmid series and transformed into wild-type *E. coli* BW25113, which is representative of a clinical isolate. AST was performed for each construct using the microdilution broth method, with the inoculum prepared using the growth method following CLSI guidelines.

Antibiotic	Resistance gene	Plasmid	MIC (μg ml^−1^) wild-type *E. coli* BW25113	CLSI resistant MIC (μg ml^−1^) breakpoint for *Enterobacteriaceae *	CLSI resistant MIC (μg ml^−1^) breakpoint for *P. aeruginosa *
**Ampicillin**	None	None	64	≥32	–
	*CMY-2*	pGDP1	>256	≥32	–
	*CTX-M-3*	pGDP1	>256	≥32	–
	*CTX-M-27*	pGDP1	>256	≥32	–
	*OXA-1*	pGDP1	>256	≥32	–
	*TEM-30*	pGDP1	>256	≥32	–
**Amoxicillin/clavulanic acid**	None	None	8–16	≥32/16	–
	*CMY-2*	pGDP1	256	≥32/16	–
	*CTX-M-3*	pGDP1	64	≥32/16	–
	*CTX-M-15*	pGDP1	16	≥32/16	–
	*OXA-1*	pGDP1	64	≥32/16	–
	*TEM-1*	pGDP1	128	≥32/16	–
**Cefazolin**	None	None	4	≥8/≥32 (urine only)	–
	*CMY-2*	pGDP1	>256	≥8/≥32 (urine only)	–
	*CTX-M-3*	pGDP1	>256	≥8/≥32 (urine only)	–
	*CTX-M-27*	pGDP1	>256	≥8/≥32 (urine only)	–
	*TEM-1*	pGDP1	256	≥8/≥32 (urine only)	–
**Cefixime**	None	None	0.25	≥4	–
	*CMY-2*	pGDP1	>256	≥4	–
	*CTX-M-3*	pGDP1	32	≥4	–
**Ceftazidime**	None	None	0.5	≥16	≥32
	*CMY-2*	pGDP1	256	≥16	nr
	*CTX-M-3*	pGDP1	16–32	≥16	nr
	*CTX-M-27*	pGDP1	128	≥16	nr
**Ertapenem**	None	None	0.25	≥2	–
	*CTX-M-27*	pGDP1	128	≥2	–
**Ceftriaxone**	None	None	0.25	≥4	–
	*CMY-2*	pGDP1	128	≥4	–
	*CTX-M-3*	pGDP1	>256	≥4	–

–, no CLSI breakpoint for *P. aeruginosa* due to intrinsic resistance; nr, not relevant as CMY-2, CTX-M-3, and CTX-M-27 were only identified in *P. aeruginosa*.

## Discussion

Fast and accurate prediction of AMR phenotypes from genotypes would improve AMR surveillance, patient outcomes and antibiotic stewardship. Currently, our ability to diagnose bacterial infections is costly and slow, contributing to the misuse and overuse of antibiotics, as well as to poor clinical outcomes. Genotypic approaches using whole-genome sequencing paired with bioinformatics resources have the potential to be a faster and more accurate method. The goal of this study was to identify and elucidate β-lactamase substrate activity, a limiting factor in AMR phenotype prediction, by using two different *in silico* AMR phenotype prediction algorithms, subsequently validated using targeted gene expression experiments. In the rules-based method, we developed EPI to be used in combination with RGI to better identify overexpressed multi-component efflux pumps, while the LR method only used the resistance determinants predicted by RGI as its starting point. While naïve about the relative contribution of individual resistance determinants to overall resistance and sensitive to any gaps in knowledge for β-lactamase activity, the rules-based method nonetheless was able to accurately predict a number of resistance phenotypes when they involved well-characterized resistance determinants that confer resistance surpassing clinical breakpoints, e.g. AAC(6′)-Ib-cr for tobramycin. In terms of false-positive predictions using this approach, we hypothesize that CARD contains incorrect genotype–phenotype information, an environmental factor is altering the expression of a predicted resistance determinant, or that CARD has a knowledge gap regarding repressors. With the first scenario, removal of incorrect curation could decrease instances of false positives, highlighting one of the limitations of human biocuration for AMR phenotype prediction. The second scenario, i.e. adaptive resistance, should not be a concern for our study, since our antibiotic susceptibility tests were standardized and automated, notwithstanding potential inconsistencies affecting gene expression [39]. The third scenario suggests that there are gaps in the literature, as CARD only includes information published in peer-reviewed literature with clear experimental evidence of elevated resistance. Genetic determinants that decrease the expression or change the substrate profile of a resistance determinant, such as mutations within regulatory regions or active sites, would result in false-positive predictions. Alternatively, entirely unknown resistance genes or mutations could explain false-negative predictions of AMR phenotypes.

To identify relationships between known resistance genes and resistance phenotypes without relying on CARD’s ARO for curated genotype–phenotype relationships, we used RGI in combination with LR. It is important to note that accurate and generalizable LR-based prediction of susceptibility or resistance to an antibiotic from detected AMR determinants is only feasible when there are relatively large numbers of genomes exemplifying each phenotype, which was not always the case in our data. Even with stratified sampling and methods, such as SMOTE [40], to resample datasets and improve balance (e.g. the relative proportion of susceptible and resistant isolates) there are limitations to what can be achieved with small datasets that are predominantly resistant or susceptible to a given antibiotic. Models that are not properly tested are likely to overfit to the data and are unlikely to generalize well for new data, in our case samples from outside the Hamilton, Ontario area. Additional validation of our models using publicly available data is important for future studies; models may be dependent on feature selection, taxonomic distribution, resistance mechanism and algorithm choice. Yet, despite the models not being appropriately tested properly due to imbalance, LR proved a useful tool for improving prediction of resistance from genomic features, even without the rules-based algorithm’s additional consideration of overexpressed, multi-component efflux pumps. LR substantially decreased instances of false positives or false negatives, and the poor performance for predicting particular resistance phenotypes (e.g. tetracycline resistance in *E. coli *, ceftazidime resistance in *P. aeruginosa* and piperacillin/tazobactam resistance in both species) could either represent a failure of the LR algorithm to capture the combination of resistance determinants required to predict resistance due to additive or synergistic resistance or to recognize undiscovered resistance determinants not in CARD and thus not predicted by RGI.

While bioinformatics tools such as breseq [41] or *k*-mer approaches combined with LR could be used to potentially identify unknown mutations or functional gene loss (e.g. OprD loss is associated with imipenem, meropenem and doripenem resistance [42]), our prediction of CLSI [43] ‘resistant’ and ‘susceptible’ resistance phenotypes places limits upon interpretation, as other clinical breakpoint guidelines exist, e.g. the European Committee on Antimicrobial Susceptibility Testing (EUCAST) [44] breakpoint guidelines are based on interpretation of quantitative MIC values, which unfortunately are not recorded in CARD or any other database for the breadth of known resistance genes and mutations. As such, detection of a CARD resistance determinant in a clinical isolate was interpreted as ‘resistant’, even though in reality the MIC value generated by the gene may not have reached the CLSI or EUCAST breakpoints for resistant. Nonetheless, aligning with George E. P. Box’s aphorism, ‘all models are wrong, but some are useful’ [45], our goal was to identify the LR models with ‘useful’ or logical biological relevance with a focus on prevalent clinical β-lactamases. Prediction of genomic determinants responsible for resistance based on the feature weights of the LR only made biological sense in some cases based on the literature and knowledge. For example, *novA* was the highest weighted predictor for *P. aeruginosa* trimethoprim/sulfamethoxazole resistance, but is known to instead be involved in the transport of and resistance to novobiocin [46]. Failure to predict logical determinants could be attributed to high levels of divergence from the canonical sequence or an unknown resistance determinant with prevalence correlated with *novA*. In the balanced datasets, known relationships in CARD, such as *tet(C*) conferring resistance to tetracycline in *E. coli* and *P. aeruginosa gyrA* mutation conferring resistance to ciprofloxacin, were predicted by both the rules-based and LR methods (Fig. 3f, n). Beyond this, LR was additionally able to predict genotype–phenotype relationships that were useful in that they were new findings not predicted by the rules-based method and not published in the literature, yet consistent with known resistance mechanisms. Indeed, there is value in looking beyond the most highly weighted LR predictor, since analysis of a model can garner major insights into AMR genotype–phenotype relationships. We were able to experimentally validate many of the top five most highly weighted candidates, illustrating that systematic screening of a broad selection of antibiotics against known resistance genes using molecular AST platforms such as the ARP [21], perhaps guided by LR, or at minimum community adoption of standard panels of antibiotics for AST characterization of newly reported resistance genes, could be adopted to fill these gaps in the literature and improve antibiotic resistance phenotype prediction.

We have illustrated that completely accurate AMR phenotype prediction is not achievable using either rules-based or LR methods. There are likely unknown genomic determinants leading to both false-positive and false-negative prediction of resistance phenotypes, such as mutations in regulatory regions that change expression of a resistance gene. Overall, our results suggest that LR is capable of predicting resistance phenotypes and identifying substrate specificities of known resistance genes when there are sufficiently balanced datasets. Evaluating learned weights for each LR model led to novel hypotheses, illustrating the use of LR as an inductive approach to guide deductive research. Yet, our results also illustrate that full prediction of resistome and resistance phenotype will require careful examination of genome feature space and clinical breakpoints, plus broad and balanced sampling of diverse susceptible and resistant strains. It is our hope that collective advances in these methods will result in tools for clinical prediction of resistance, aiding antimicrobial stewardship and improving patient outcomes. Elucidating AMR genotype–phenotype relationships will reveal the genetic and mechanistic underpinnings of resistance to guide both public health surveillance and future drug discovery.

## Methods

### Bacterial isolates, antibiotic susceptibility testing, and DNA extraction

Clinical bacterial isolates were obtained from the IIDR Clinical Isolate Collection, which consists of isolates from the core clinical laboratory at Hamilton Health Sciences, Hamilton, Ontario. Samples were collected between 2015 and 2018 and were resistant to 3 or more antibiotics based on antimicrobial susceptibility to 18 and 17 antibiotics for *E. coli* and *P. aeruginosa *, respectively. As ertapenem lacks activity against *P. aeruginosa* [47], it was not included in *P. aeruginosa* antibiotic susceptibility tests. Initial culture and antibiotic susceptibility testing (AST) were performed by Hamilton General Hospital General Microbiology Laboratory using a VITEK 2 Automated System and its Advanced Expert System (BioMérieux, Marcy-l′Étoile, France), compliant with the Clinical and Laboratory Standards Institute (CLSI) [43] antibiotic susceptibility testing formulations, reporting CLSI breakpoint-determined susceptible (S), intermediate (I), or resistant (R). For DNA extraction, isolates were provided on blood agar plates and single colonies were restreaked onto brain heart infusion (BHI) agar. After overnight incubation, single colonies of each isolate were used to inoculate Luria–Bertani (LB) broth. Overnight broth cultures were used to prepare glycerol stocks for long-term storage at −80 °C. One millilitre of the same overnight cultures was centrifuged, the supernatant was removed and the pellet was stored at −80 °C for genomic DNA extraction. The Invitrogen Pure Link Genomic DNA Mini kit (K182002) was used for DNA extraction from pellets. DNA was eluted with water and stored at 4 °C.

### Whole-genome sequencing, assembly and species identification

DNA sequencing library construction (Illumina Nextera XT DNA Library Preparation kit or NEBNext Ultra II DNA Library Preparation kit) and all sequencing runs were performed at the Farncombe Metagenomics Facility at McMaster University using 2×150 bp paired-end sequencing on an Illumina HiSeq 1500 platform (*E. coli n*=115, *P. aeruginosa n*=92) or 2×250 bp paired-end sequencing on an Illumina MiSeq v3 platform (*P. aeruginosa n*=10). Paired sequencing reads were trimmed using Trimmomatic (v0.36) [48], checked for quality using fastqc (v0.11.8, http://www.bioinformatics.babraham.ac.uk/projects/fastqc/) [49] and *de novo* assembled using SPAdes (v3.9.0) [50]. The Livermore Metagenomics Analysis Toolkit (lmat, v1.2.6) [51] was used to confirm bacterial species and screen for contamination or mixed culture. For *E. coli *, after quality trimming of the sequencing reads by Trimmomatic, sequencing isolate read coverage averaged 207.5-fold, assembly size averaged ~5 163 879 bp and N50s averaged 231 879 bp. For *P. aeruginosa,* quality-trimmed sequencing read coverage averaged 100.6-fold, assembly sizes averaged 6 680 703 bp and assembly N50s averaged 260 849 bp. Diversity of isolates for both *E. coli* and *P. aeruginosa* was assessed by multilocus sequence typing (MLST) via comparison to the reference sequences available at pubMLST (https://github.com/agmcarthur/pubMLST) [52].

### Curation of CARD

At minimum, CARD requires the curation of a ‘*confers_resistance_to_drug_class*’ relationship between an AMR gene family and a drug class in the ARO. However, to predict specific drug resistance phenotypes we needed curation of a ‘*confers_resistance_to_antibiotic*’ relationship between an individual resistance gene or mutation and a specific antibiotic. The curation of ‘*confers_resistance_to_antibiotic*’ relationships is incomplete in CARD and is determined by experimental evidence of an elevation of MIC in the published literature [7]. Using extensive literature review, we curated ‘*confers_resistance_to_antibiotic*’ relationships for all resistance determinants identified as RGI Perfect or Strict RGI hits for our *E. coli* and *P. aeruginosa* isolates: an additional 250 ‘*confers_resistance_to_antibiotic*’ relationships (152 *E. coli* and 98 *P *. *aeruginosa *) were added to CARD (available as of v2.0.2). During the curation process we also identified two errors in CARD curation. These included incorrect inclusion of mutation Y45C in the *E. coli* protein *NfsA* as conferring resistance to nitrofurantoin and the β-lactamase gene *SHV-1* as conferring resistance to cefazolin. In both cases, the original publications lacked clear experimental support for these claims.

To additionally improve efflux pump prediction and facilitate the functionality of the Efflux Pump Identifier (EPI), *E. coli* and *P. aeruginosa* efflux meta-models (a combination of individual models) were curated into CARD v1.1.9, based on review of the literature. Efflux meta-models comprise protein homologue and/or protein overexpression models to represent a known efflux pump complex and its regulatory network. For example, the *AcrAB-TolC* efflux system (ARO:3000384) is encoded along with its regulatory network: *marR*, *marA*, *acrR*, *sdiA*, *soxS*, *soxR*, *rob*. In this meta-model, each component is a protein homologue model with the exception of *marR*, *acrR* and *soxS*, which are protein overexpression models. We curated 21 *P *. *aeruginosa* efflux pump meta-models, 10 *E. coli* efflux pump meta-models and 2 plasmid-borne efflux pump meta-models known to confer resistance to the 18 antibiotics tested in this study for analysis by EPI.

### Rules-based prediction of antibiotic susceptibility phenotypes

Isolate genomes were analysed using the Comprehensive Antibiotic Resistance Database (v2.0.2) and Resistance Gene Identifier (v4.1.0) [7], plus the new EPI (v1.0.0) software developed by KKT, to predict resistance determinants. The EPI predicts multi-component efflux pumps and their regulatory networks using the efflux meta-models curated in CARD (https://git.io/JJFhT). RGI and EPI results were filtered to only include RGI Perfect and Strict hits, and EPI Perfect and Partial hits, respectively. Antibiotic susceptibility phenotypes were predicted by traversing CARD’s Antibiotic Resistance Ontology (ARO) to identify the antibiotic(s) each detected resistance determinant confers resistance to, based on peer-reviewed literature. In this rules-based method, the detection of a resistant determinant by RGI or EPI that had a ‘*confers_resistance_to_antibiotic*’ relationship to an antibiotic in the ARO resulted in a ‘resistant’ phenotype prediction, otherwise a ‘susceptible’ phenotype was predicted. Computational antibiotic susceptibility predictions were then compared to clinical ASTs. As AST ‘intermediate’ resistances were rare (2.2 % of *P. aeruginosa* resistance phenotypes and 3.6 % of *E. coli* resistance phenotypes), we treated them as ‘resistant’ in our analyses.

### Using logistic regression to predict antibiotic resistance phenotypes

To prepare the datasets, all RGI results for each species were collated into count matrices *X*_*ij*_ where *i* represents each genome of that species and *j* represents a specific AMR determinant detected by RGI at either Strict or Perfect cut-offs. The most appropriate algorithm for phenotype prediction was determined using the *E. coli* data, as these comprised the more balanced dataset. For each antibiotic, the resampled training data were used to fit four interpretable binary classification models: logistic regression, multinomial naïve Bayes, decision tree and random forest classifiers [53]. For each model the hyperparameters were then tuned using a threefold stratified shuffle split cross-validation scheme and evaluated using a negative log loss scoring function [53], as negative log loss considers prediction uncertainty in relation to the divergence of the predicted probabilities and the actual AMR phenotype. Logistic regression and random forest classifiers had the highest performance of all tested modelling methods, so we chose logistic regression, a simpler algorithm, as our classification paradigm under the principle of parsimony. To predict each antibiotic resistance phenotype, antibiotic-specific LR models were trained, optimized via cross-validation and tested separately for each species dataset. To determine whether each species and antibiotic dataset was phenotypically balanced enough for LR, the relative proportion of resistant predictions to susceptible predictions was evaluated. If the less frequent phenotype represented <10 % of all genomes it was considered inappropriate to train and properly test a model due to extreme class imbalance and low signal. For these antibiotics an ‘unbalanced classifier’ was trained and evaluated using all genomes of that species. Some antibiotics displayed an even more extreme case of imbalance where only a single phenotype was observed. For these, a ‘dummy’ model was used that only returned the observed phenotype (i.e. all observed isolates were resistant to an antibiotic and therefore the model always predicts resistance). For the remaining species-antibiotics combinations with greater label balance, 20 % of the genomes were randomly selected with stratification (i.e. maintaining the relative proportion of susceptible to resistant) and withheld as a test set. The training set was then rebalanced using the synthetic minority over-sampling technique (SMOTE) [40] as implemented in imbalanced-learn (v0.3.3) [54] to generate a training set with equal proportions of susceptible and resistant genomes. After training of the *E. coli* models, the *P. aeruginosa* training data were used to fit and optimize logistic regression models via the same threefold stratified cross-validation scheme.

The individual trained antibiotic–species logistic regression models (including unbalanced and dummy classifiers) were evaluated against the test set to see if they could predict AMR phenotype, with evaluation using precision–recall curves (summarized as average precision) and the receiver operating characteristic (summarized as area under the curve) (Figs S1–S3) [55]. A test with perfect discrimination between resistance and susceptible resistance phenotypes would have a receiver operating characteristic curve that passes through the upper-left corner (Figs S1 and S2). For each species the number of true positives, true negatives, false positives and false negatives was tallied and plotted for each antibiotic. To evaluate which resistance determinants within each classifier were important for predicting resistance phenotypes, we considered the estimated coefficients (scikit-learn’s coef_attribute) as the ‘weight of importance’ for each resistance determinant. Thus, given two resistance determinants, each with an estimated coefficient value, the resistant determinant with a larger estimated coefficient value was interpreted as more important for predicting a particular resistance phenotype. The five most highly weighted predictors of each resistance phenotype were examined (Figs S4 and S5), but all feature weights of importance and their *P*-values were inspected and are listed in Tables S2–S5.

### Antibiotic susceptibility testing (AST) using the Antibiotic Resistance Platform

In cases where we wished to perform AST for individual resistance genes, we cloned these genes into pGDP1/pGDP3 from the Antibiotic Resistance Platform [21] and transformed into wild-type *E. coli* BW25113. AST was performed for *E. coli* BW25113 using the microdilution broth method, with the inoculum prepared using the growth method following CLSI guidelines [43]. Plates were sealed in a bag and incubated for 18 h at 37 °C, 250 r.p.m. before the optical density at 600 nm was measured using the Spectramax microplate reader.

### Software availability

CARD data and RGI software are available at the CARD website, http://card.mcmaster.ca. CARD (v2.0.2) and RGI (v4.1.0) were used for all resistome prediction, and RGI (v.5.1.0) was used for creating the heatmaps. The EPI software is available at https://github.com/karatsang/rulesbased_logisticregression/tree/v1.0.0/rulesbased/EffluxPumpIdentifier. LR and dataset partitioning were performed using scikit-learn (v0.20.0) [53] with data otherwise manipulated using numpy (v1.17.2) [56] and pandas (v0.25.1) [57]. For both datasets, the code, conda environments (using python v3.7.2 [58]), and intermediate data files required to generate this analysis are available: https://github.com/karatsang/rulesbased_logisticregression, https://doi.org/10.5281/zenodo.3988480.

## Supplementary Data

Supplementary material 1

Supplementary material 2
