# Supplementary material for: Identifying novel β-lactamase substrate activity through in silico prediction of antimicrobial resistance
Source: Microb Genom. 2021 Jan 8;7(1):mgen000500. doi: 10.1099/mgen.0.000500 (PMC8115898; doi:10.1099/mgen.0.000500)
Supplement: Supplementary material 1 [file mgen-7-500-s001.pdf]

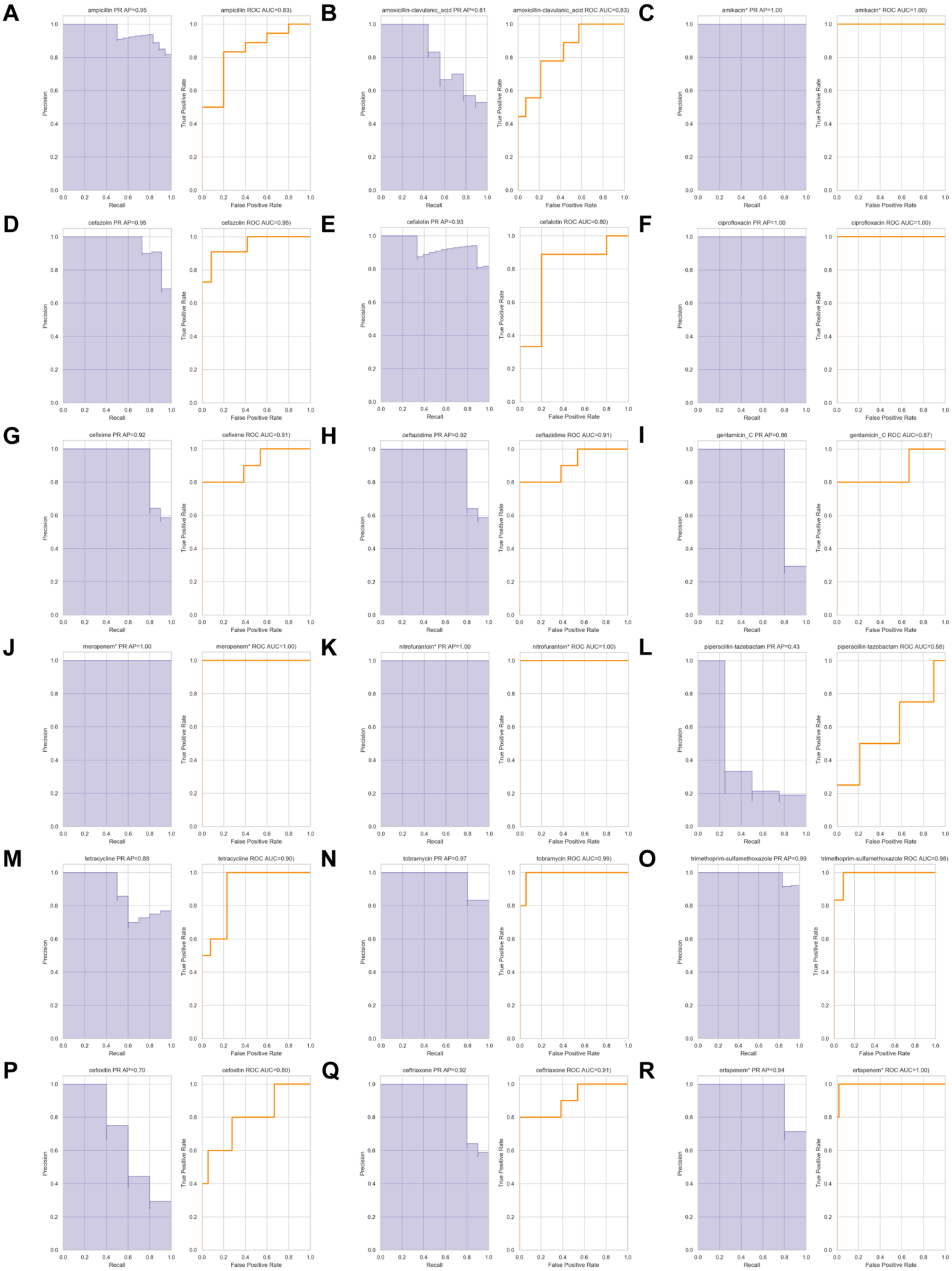
A
ampicillin PR AP=0.95
ampicillin ROC AUC=0.83)
B
amoxicillin-clavulanic_acid PR AP=0.81
amoxicillin-clavulanic_acid ROC AUC=0.83)
C
amikacin* PR AP=1.00
amikacin* ROC AUC=1.00)
D
cefazolin PR AP=0.95
cefazolin ROC AUC=0.95)
E
cefalotin PR AP=0.93
cefalotin ROC AUC=0.80)
F
ciprofloxacin PR AP=1.00
ciprofloxacin ROC AUC=1.00)
G
cefixime PR AP=0.92
cefixime ROC AUC=0.91)
H
ceftazidime PR AP=0.92
ceftazidime ROC AUC=0.91)
I
gentamicin_C PR AP=0.86
gentamicin_C ROC AUC=0.87)
J
meropenem* PR AP=1.00
meropenem* ROC AUC=1.00)
K
nitrofurantoin* PR AP=1.00
nitrofurantoin* ROC AUC=1.00)
L
piperacillin-tazobactam PR AP=0.43
piperacillin-tazobactam ROC AUC=0.58)
M
tetracycline PR AP=0.88
tetracycline ROC AUC=0.90)
N
tobramycin PR AP=0.97
tobramycin ROC AUC=0.99)
O
trimethoprim-sulfamethoxazole PR AP=0.99
trimethoprim-sulfamethoxazole ROC AUC=0.98)
P
cefoxitin PR AP=0.70
cefoxitin ROC AUC=0.80)
Q
ceftriaxone PR AP=0.92
ceftriaxone ROC AUC=0.91)
R
ertapenem* PR AP=0.94
ertapenem* ROC AUC=1.00)
Precision
Recall
True Positive Rate
False Positive Rate

Figure S1. Precision-Recall (PR) and Receiver Operating Characteristic (ROC) curves for logistic regression models developed for *E. coli* antibiotic resistance phenotype prediction. Models for (A) ampicillin, (B) amoxicillin-clavulanic acid, (C) amikacin, (D) cefazolin, (E) cefalotin, (F) ciprofloxacin, (G) cefixime, (H) ceftazidime, (I) gentamicin, (J) meropenem, (K) nitrofurantoin, (L) piperacillin-tazobactam, (M) tetracycline, (N) tobramycin, (O) trimethoprim-sulfamethoxazole, (P) cefoxitin, (Q) ceftriaxone, (R) ertapenem which <10% of a species' isolates displayed susceptible or resistant phenotypes could not be properly validated and tested (4 antibiotics for *E. coli*), so were trained using all the data (indicated by an asterisk).

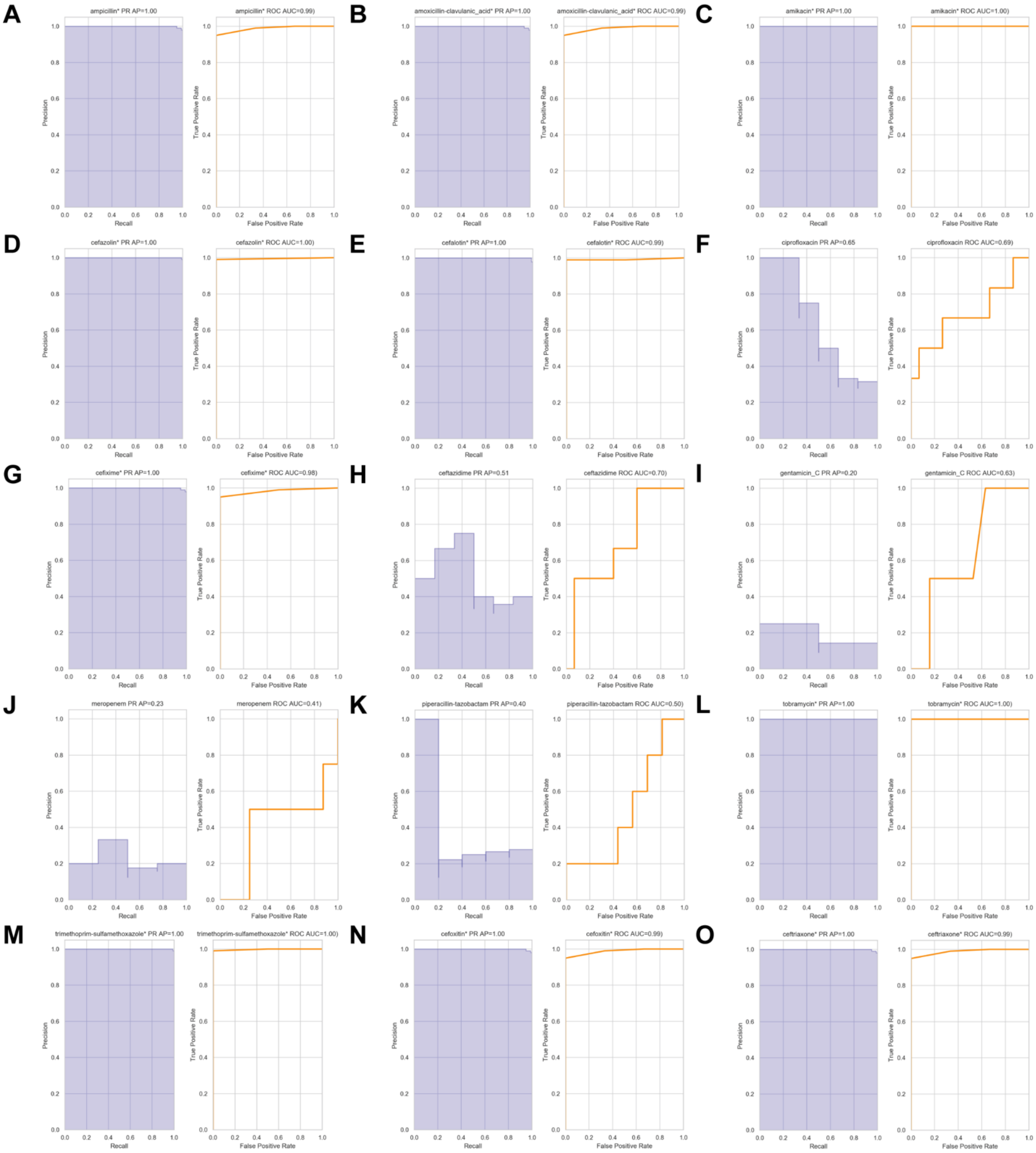
A
ampicillin* PR AP=1.00
ampicillin* ROC AUC=0.99)
B
amoxicillin-clavulanic_acid* PR AP=1.00
amoxicillin-clavulanic_acid* ROC AUC=0.99)
C
amikacin* PR AP=1.00
amikacin* ROC AUC=1.00)
D
cefazolin* PR AP=1.00
cefazolin* ROC AUC=1.00)
E
cefalotin* PR AP=1.00
cefalotin* ROC AUC=0.99)
F
ciprofloxacin PR AP=0.65
ciprofloxacin ROC AUC=0.69)
G
cefixime* PR AP=1.00
cefixime* ROC AUC=0.98)
H
ceftazidime PR AP=0.51
ceftazidime ROC AUC=0.70)
I
gentamicin_C PR AP=0.20
gentamicin_C ROC AUC=0.63)
J
meropenem PR AP=0.23
meropenem ROC AUC=0.41)
K
piperacillin-tazobactam PR AP=0.40
piperacillin-tazobactam ROC AUC=0.50)
L
tobramycin* PR AP=1.00
tobramycin* ROC AUC=1.00)
M
trimethoprim-sulfamethoxazole* PR AP=1.00
trimethoprim-sulfamethoxazole* ROC AUC=1.00)
N
cefoxitin* PR AP=1.00
cefoxitin* ROC AUC=0.99)
O
ceftriaxone* PR AP=1.00
ceftriaxone* ROC AUC=0.99)
Precision
Recall
True Positive Rate
False Positive Rate

Figure S2. Precision-Recall (PR) and Receiver Operating Characteristic (ROC) curves for logistic regression models developed for *P. aeruginosa* antibiotic resistance phenotype prediction. Models for (A) ampicillin, (B) amoxicillin-clavulanic acid, (C) amikacin, (D) cefazolin, (E) cefalotin, (F) ciprofloxacin, (G) cefixime, (H) ceftazidime, (I) gentamicin, (J) meropenem, (K) piperacillin-tazobactam, (L) tobramycin, (M) trimethoprim-sulfamethoxazole, (N) cefalotin, (O) ceftriaxone resistance which <10% of a species' isolates displayed susceptible or resistant phenotypes could not be properly validated and tested (10 antibiotics for *P. aeruginosa*), so were trained using all the data (indicated by an asterisk). Tetracycline, nitrofurantoin, and ertapenem resistance prediction models could not be developed for the following reasons. All isolates were resistant to tetracycline and nitrofurantoin, thus a 'dummy' model was used which always returns the relevant label. Ertapenem phenotypic AST was not performed for *P. aeruginosa.*

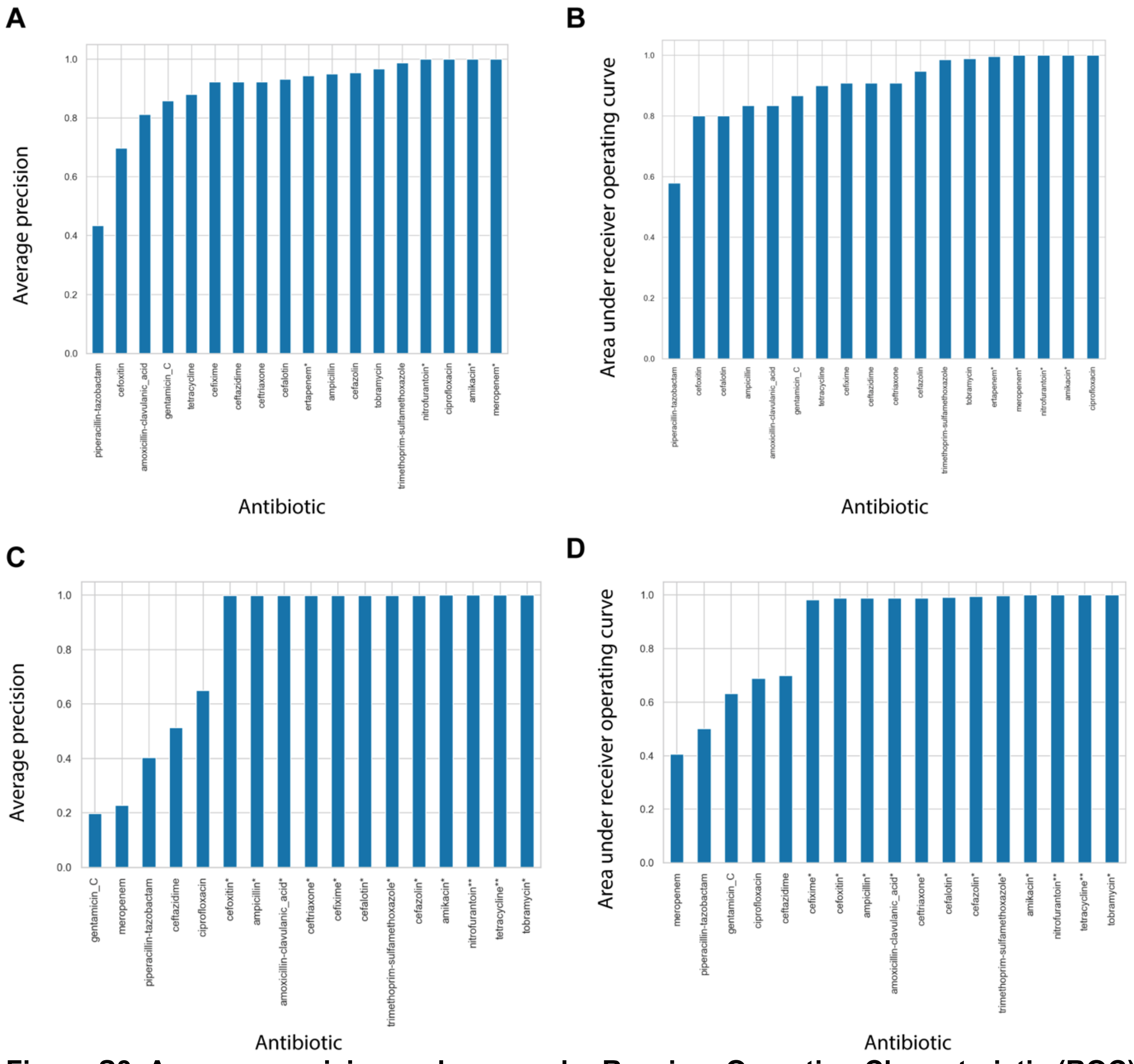

Figure S3. Average precision and area under Receiver Operating Characteristic (ROC) graphs for (A, B) *E. coli* and (C, D) *P. aeruginosa* logistic regression models used for resistance phenotype prediction. X-axis indicates the antibiotic tested whereas the y-axis indicates the (A, C) average precision or the (B, D) area under the ROC curve for each logistic regression model. Models for antibiotics for which <10% of a species' isolates displayed susceptible or resistant phenotypes could not be properly validated and tested (10 antibiotics for *P. aeruginosa* and 4 antibiotics for *E. coli*), so were trained using all the data (indicated by an asterisk).

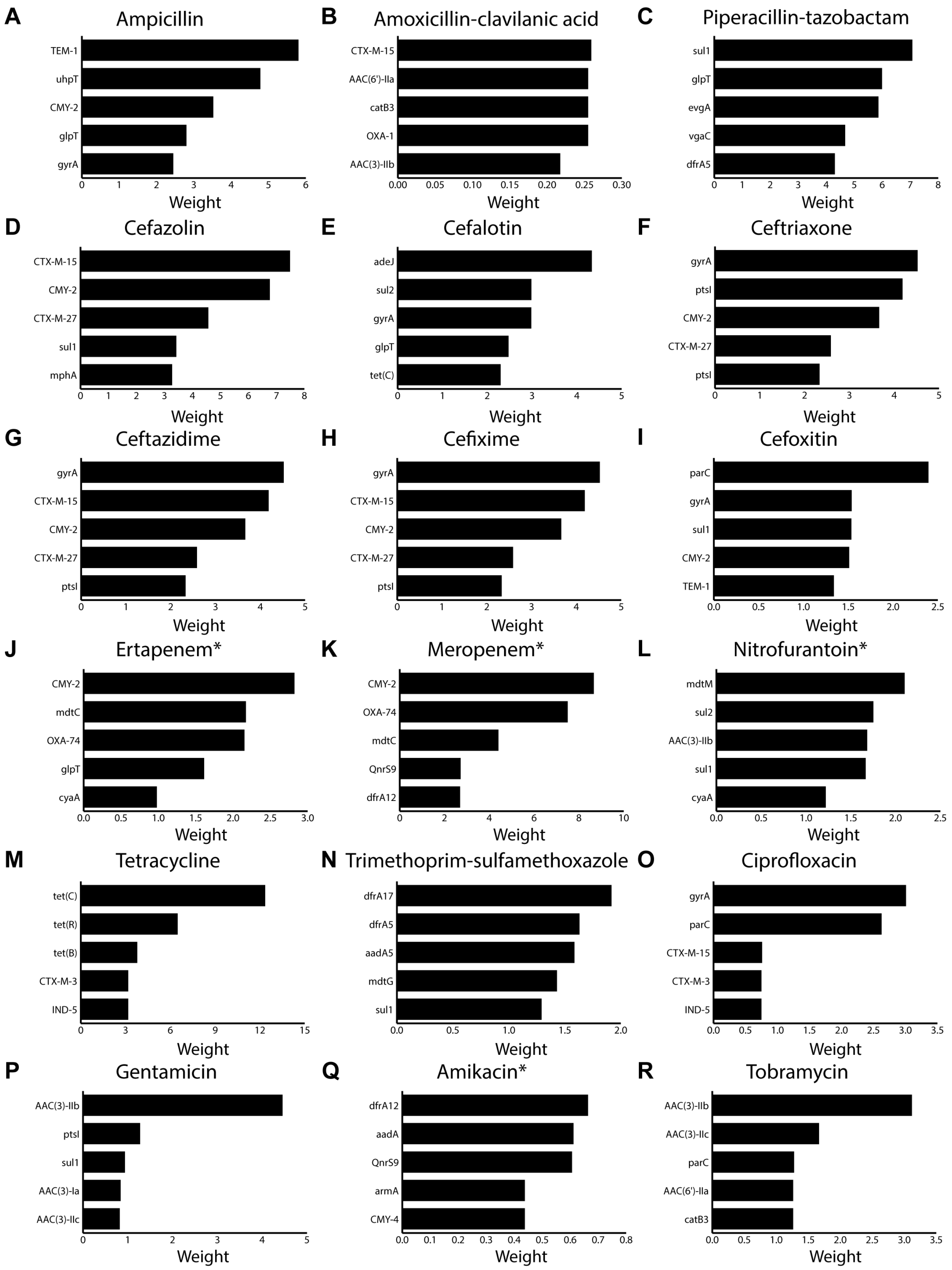

A
Ampicillin
TEM-1
uhpT
CMY-2
glpT
gyrA
Weight
B
Amoxicillin-clavilanic acid
CTX-M-15
AAC(6')-IIa
catB3
OXA-1
AAC(3)-IIb
Weight
C
Piperacillin-tazobactam
sul1
glpT
evgA
vgaC
dfrA5
Weight
D
Cefazolin
CTX-M-15
CMY-2
CTX-M-27
sul1
mphA
Weight
E
Cefalotin
adeJ
sul2
gyrA
glpT
tet(C)
Weight
F
Ceftriaxone
gyrA
ptsI
CMY-2
CTX-M-27
ptsI
Weight
G
Ceftazidime
gyrA
CTX-M-15
CMY-2
CTX-M-27
ptsI
Weight
H
Cefixime
gyrA
CTX-M-15
CMY-2
CTX-M-27
ptsI
Weight
I
Cefoxitin
parC
gyrA
sul1
CMY-2
TEM-1
Weight
J
Ertapenem*
CMY-2
mdtC
OXA-74
glpT
cyaA
Weight
K
Meropenem*
CMY-2
OXA-74
mdtC
QnrS9
dfrA12
Weight
L
Nitrofurantoin*
mdtM
sul2
AAC(3)-IIb
sul1
cyaA
Weight
M
Tetracycline
tet(C)
tet(R)
tet(B)
CTX-M-3
IND-5
Weight
N
Trimethoprim-sulfamethoxazole
dfrA17
dfrA5
aadA5
mdtG
sul1
Weight
O
Ciprofloxacin
gyrA
parC
CTX-M-15
CTX-M-3
IND-5
Weight
P
Gentamicin
AAC(3)-IIb
ptsI
sul1
AAC(3)-Ia
AAC(3)-IIc
Weight
Q
Amikacin*
dfrA12
aadA
QnrS9
armA
CMY-4
Weight
R
Tobramycin
AAC(3)-IIb
AAC(3)-IIc
parC
AAC(6')-IIa
catB3
Weight

Figure S4. The top five highest weights of importance for *E. coli* antibiotic resistance phenotype prediction. The x-axis indicates assigned LR weights for individual antibiotics, while the y-axis list the top five weighted AMR determinants. Models for (A) ampicillin, (B) amoxicillin-clavulanic acid, (C) piperacillin-tazobactam, (D) cefazolin, (E) cefalotin, (F) ceftriaxone, (G) ceftazidime, (H) cefixime, (I) cefoxitin, (J) ertapenem, (K) meropenem, (L) nitrofurantoin, (M) tetracycline, (N) trimethoprim-sulfamethoxazole, (O) ciprofloxacin, (L) gentamicin (Q) amikacin, (R) tobramycin resistance which <10% of a species' isolates displayed susceptible or resistant phenotypes could not be properly validated and tested (4 antibiotics for *E. coli*), so were trained using all the data (indicated by an asterisk).

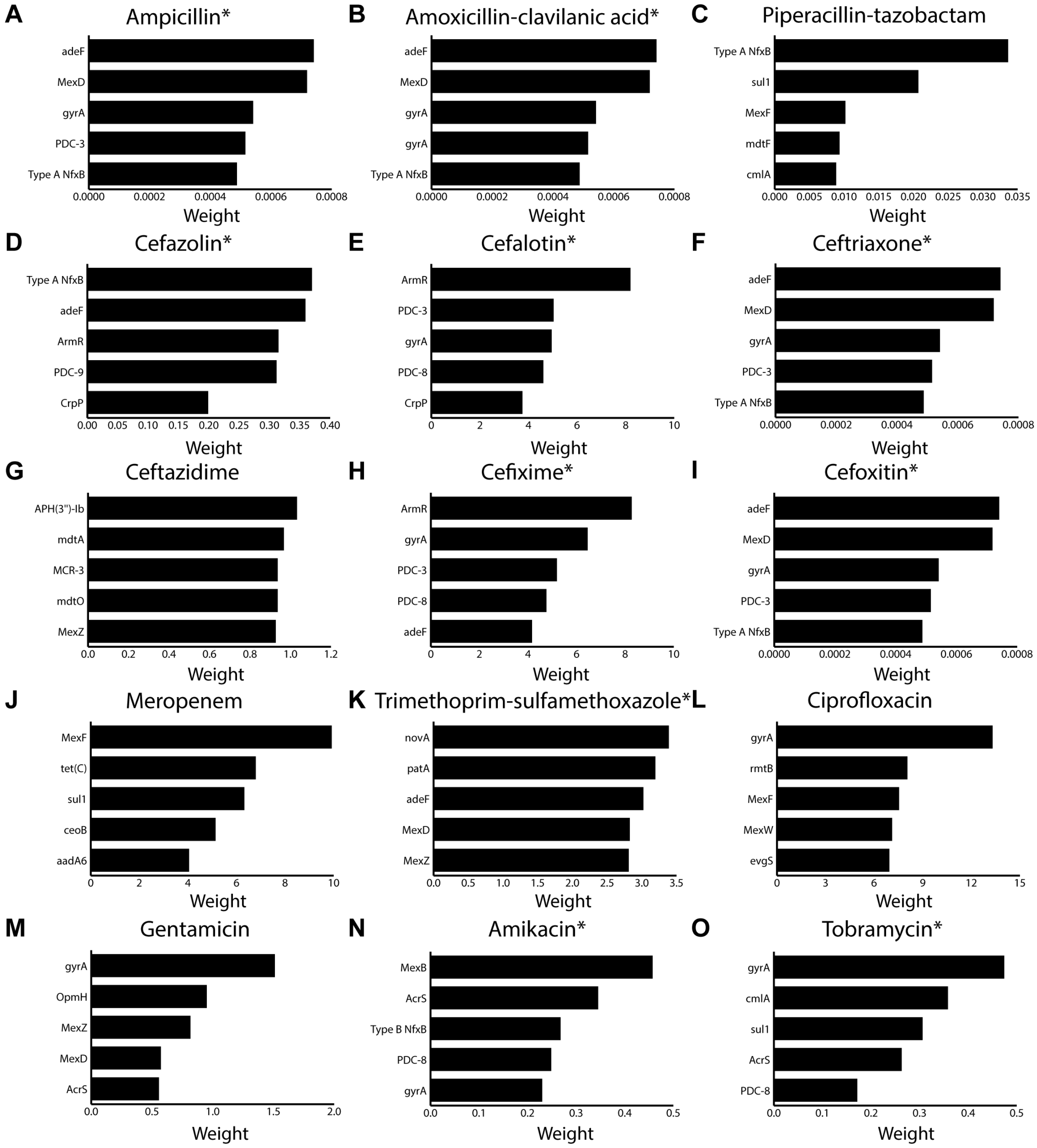

Figure S5. The top five highest weights of importance for *P. aeruginosa* antibiotic resistance phenotype prediction. The x-axis indicates assigned LR weights for individual antibiotics, while the y-axis list the top five weighted AMR determinants. Models for (A) ampicillin, (B) amoxicillin-clavulanic acid, (C) piperacillin-tazobactam, (D) cefazolin, (E) cefalotin, (F) ceftriaxone, (G) ceftazidime, (H) cefixime, (I) cefoxitin, (J) meropenem, (K) trimethoprim-sulfamethoxazole, (L) ciprofloxacin, (M) gentamicin, (N) amikacin, (O) tobramycin resistance which <10% of a species' isolates displayed susceptible or resistant phenotypes could not be properly validated and tested (10 antibiotics for *P. aeruginosa*), so were trained using all the

data (indicated by an asterisk). Tetracycline, nitrofurantoin, and ertapenem resistance prediction models could not be developed for the following reasons. All isolates were resistant to tetracycline and nitrofurantoin, thus a ‘dummy’ model was used which always returns the relevant label. Ertapenem phenotypic AST was not performed for *P. aeruginosa.*

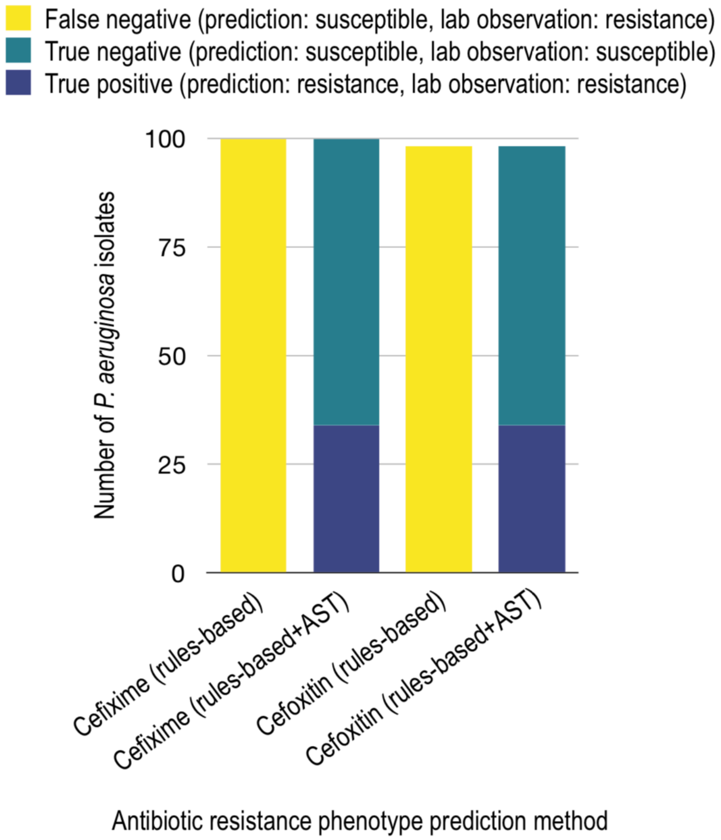

Figure S6. Improvement of *P. aeruginosa* cefixime and cefoxitin resistance prediction using information gained from ASTs, RGI and ARO. Through antibiotic susceptibility testing (AST), we observed PDC-3 and PDC-5 conferring resistance to cefixime and cefoxitin. Curating this knowledge into CARD would improve cefixime and cefoxitin resistance true positive prediction in *P. aeruginosa* by 34.0% and 34.7%, respectively. However, there are no CLSI breakpoint guidelines for cefixime and cefoxitin because they are not used clinically to treat *P. aeruginosa* infections.
